# Knowledge about COVID-19 between Children and Adolescents with and without High Intellectual Abilities

**DOI:** 10.3390/healthcare11172408

**Published:** 2023-08-28

**Authors:** Gabriela López-Aymes, María de los Dolores Valadez Sierra, África Borges, Grecia Emilia Ortiz Coronel, Juan Francisco Flores-Bravo, Celia Josefina Rodríguez-Cervantes, Norma A. Ruvalcaba-Romero

**Affiliations:** 1 Transdisciplinary Research Center in Psychology, Autonomous University of the State of Morelos, Cuernavaca 62350, Mexico; 2 Institute of Psychology and Special Education, Department of Applied Psychology, University Center for Health Sciences, University of Guadalajara, Guadalajara 44340, Mexico; grecia.ortiz5850@academicos.udg.mx (G.E.O.C.); juan.flores8719@academicos.udg.mx (J.F.F.-B.); norma.ruvalcaba@academicos.udg.mx (N.A.R.-R.); 3 Department of Clinical Psychology, Psychobiology and Methodology, University of La Laguna, 38200 San Cristóbal de La Laguna, Spain; aborges@ull.edu.es; 4 National System for Integral Family Development, Tlajomulco de Zúñiga 45640, Mexico; celiajo_1@hotmail.com

**Keywords:** knowledge, COVID-19, children, adolescents, gifted people

## Abstract

Among the characteristics within people with high intellectual abilities, some that stand out are a better handling of information, asynchronous development, high awareness, and sensibility. Therefore, our goal was to learn if, due to these characteristics, the children and adolescents with high intellectual abilities have a better understanding and comprehension about COVID-19 compared to those with average intellectual abilities. A qualitative study was conducted at the beginning of the lockdown with 649 children with and without high intellectual abilities. An online questionnaire was used and three open questions were analyzed with the ALCESTE software. The results showed that both groups had a similar handling of the information regarding COVID-19. Despite this, in the high ability group there is a greater social concern, which coincides with some characteristics associated with a more developed moral conscience. The results are then discussed in terms of the importance of designing actions that allow us to adequately follow the control and intervention strategies, as well as to propose improvements in the communication of relevant information before diverse crises to which the child population may be exposed.

## 1. Introduction

The year 2020 has been marked forever in the memory of humanity. The COVID-19 pandemic, which was declared by the World Health Organization [WHO] on 11 March 2020 [1], spread over five continents, paralyzing some of the essential functions and activities of individuals and disrupting daily life, including education. By the 25th epidemiological week, more than two million cumulative cases of infections were reported in Mexico, including deaths associated with the clinical diagnosis of COVID-19, so pandemic control measures had to be prolonged for a long time [2]. The COVID-19 pandemic caused the closure of all facilities, causing people to stay in their homes [3]. Among the prevention measures that were implemented at the national level based on WHO recommendations were constant hand washing, sneezing etiquette, avoiding touching eyes, nose, and mouth, using masks, healthy distancing, staying at home, sanitization, and isolation; in addition, community control filters were installed. In this way, the reopening of activities was carried out in a gradual, orderly and cautious manner, in order to control the transmission of the SARS-CoV-2 virus [4]. The lockdown had an impact in Mexico, increasing social vulnerability. For example, the education system was affected by a prolonged closure of its facilities and a move to virtual or distance education (from March 2020 to September 2021), which is likely to cause pre-existing inequalities to widen [5]. The pandemic provided a new opportunity for the use of digital media. During the lockdown, digital media consumption increased by 65–75% in many countries around the world [6]. However, the overabundance of information on this phenomenon, whether accurate or false, known as infodemics, provoked various behaviors from the population, ranging from compliance with protective measures dictated by the government, to xenophobic events, aggressions against health personnel, and denial of the disease [7]. This of course led to difficulties in finding reliable information [8], and misinformation about COVID-19 has claimed lives around the world [9].

Although children and adolescents have not been the most affected groups in terms of health by the virus, they are a vulnerable group since the measures taken by the government have altered their life and their habits [10,11]. A survey conducted by the United Nations Children’s Fund (UNICEF) highlights that the collateral effects of the lockdown on children included individual factors (nutrition, physical and mental health), as well as context factors (education, protection, leisure and recreation), because children have been exposed to adverse conditions like mistreatment, violence, abuse, or exploitation. Moreover, they have been continuously exposed to information from their primary sources like parents and traditional and social media [1]. However, the consequences of the lockdown on both children and young people have yet to be studied, so more research is needed. According to the “Web of the Science”, accessed in January 2022, only 1093 out of 138,399 articles addressed emotions (0.78%), of which only 110 addressed children; three of these focused on children with special educational needs and only one on children with high intellectual abilities. Most of these studies were parental reports of their children, in agreement with what Martiny et al. [12] pointed out, that empirical research on children’s perception, its restrictions, and the effect of the pandemic on their wellbeing is still scarce.

Given the lack of knowledge of the health risk associated with COVID-19, there were multiple perceptions on how to face it; the most evidence on risk perception comes from research in previous pandemics, especially the H1N1 Swine Flu in 2009, the Ebola outbreak, as well as the SARS and the Avian Flu epidemics [13]. In the case of COVID-19, the perception can be shaped by media pressure or life experience, but in this case, there was no previous experience of the same magnitude [14]. It is important to know the perception of adults and their self-care practices within their parenting role, as they are the decision makers at home and if their knowledge is mistaken, the health of the young population will be at risk [7,15,16].

The knowledge and perceptions from children and adolescents regarding COVID-19 are important because it is known that the perception they have of the restrictions will have an impact on their wellbeing [12]. Jiao et al. [17] mention that in order to give appropriate attention to children and adolescents’ emotions, it is important to know and understand first their reactions and emotions. Bonoti et al. [18] state that it is fundamental to know how children understand COVID-19 in order to develop targeted campaigns focused on their protection.

In the research by Idoiaga et al. [19], the authors identified the social and emotional representations from kids regarding COVID-19 through an analysis of their answers with the Reinet method using Iramuteq software for lexical analysis; this method is based on the premise that all discourses are expressed through groups of words that can be organized relationally. From this approach, the situations, thoughts, and emotions present in the lexical worlds or social representations shared by school children in schools in a region of northern Spain were examined [19]. The researchers identified that although the children represent COVID-19 as a harming “bug”, they also acknowledge the battle of the physicians against it. Regarding their emotions, concern and fear were observed on the one hand, and safety and joy of being with their families on the other hand. These results demonstrate the need to address the effects of the pandemic on children.

In the study conducted by Cauberghe et al. [20] on children during the COVID-19 lockdown, it was found that the period of confinement and the threat of the disease caused concern, such as getting sick from the virus; as for the educational context, they expressed concerns related to the lack of school content, that is, the absence or insufficiency of educational subjects that allow for the fulfillment of the curriculum, as well as the delay in planning.

Regarding the knowledge that children have about COVID-19, it has been found that they identify the causes and symptoms of COVID-19, as well as the importance of precautionary measures [18].

In a study carried out in Spain, which sought to find out how children were experiencing the lockdown, in which 425 children and adolescents between 8 and 17 participated, resilience was present in these children, but at a high cost in terms of emotions and reactions to the pandemic [21]. In another study carried out by 47 organizations related to social work with children in Central America and Mexico, it was found that COVID-19 caused them sadness, worry, and fear [22]. These results are consistent with those obtained in other studies conducted with children [23,24,25].

Few studies have been conducted with children with special needs, among which the following stand out: those conducted with children and adolescents with attention-deficit/hyperactivity disorder [26,27,28], autistic spectrum disorder [29,30], or with high intellectual abilities [31], among other needs. Regarding children and adolescents with high intellectual abilities, Amend et al. [32] mention that due to their characteristics, they may experience anxiety or intense emotions when facing COVID-19. These characteristics, such as asynchronous development and high perception and sensitivity, can intensify their reactions to events and, combined with external factors, can jeopardize their social and emotional well-being [33,34]. It is true that they can cognitively process information about COVID-19, but for some, their emotional or social skills may not be as mature. When exposed to inaccurate information or media, high-ability children may imagine situations that are more frightening than reality [33]. A study by Valadez et al. [25] found that there were no significant differences in reactions and emotions to COVID-19 during the first months of lockdown between children and adolescents with high intellectual abilities, and those in a community sample. Duraku and Hoxha [35], on the other hand, found a negative impact on the psychological well-being of children with high intellectual abilities, in the face of social isolation and school closures due to COVID-19.

Given these characteristics associated with people with high intellectual abilities, we are wondering whether students with high intellectual abilities have a greater knowledge and understanding of this new virus compared to students of average abilities; in addition, we are interested in knowing if there are differences in the perception of the consequences of the disease and self-care behaviors, compared to students without these characteristics. 

Therefore, the objective of this research is to analyze the knowledge about COVID-19 among children and adolescents with and without high intellectual abilities, and to compare if there are differences between these groups, since these factors allow us to adequately follow the control and intervention strategies, as well as to propose improvements in the communication of relevant information before diverse crises to which the child population may be exposed.

## 2. Materials and Methods

### 2.1. Participants

A convenience sampling procedure was used to determine the sample. It consisted of 649 children between 5 and 14 years of age (mean = 9.6, SD = 2.6). A total of 319 belonged to the population with high intellectual abilities (115 girls and 204 boys) and 330 belonged to the community sample (155 girls and 175 boys). Most of them were from Mexico (624), and the rest from Spain (20) and the United States (2). In the case of children between 5 and 7 years of age who had not acquired reading skills, they could rely on their parents for reading the items. Table 1 shows the characteristics of the participants.

### 2.2. Instruments

An instrument of 7 open-ended questions was designed, distributed in two sections, namely:

(1) General data, where age, sex, type of school attended, participation in psychoeducational intervention programs, and place of residence are explored.
(2) Knowledge and attitudes towards the health contingency caused by COVID-19. The items that compose the questionnaire are described in Table 2.

In this study, only the first three items of the instrument were considered, since these are the ones that refer to knowledge about the COVID-19 pandemic. 

### 2.3. Procedure

The research group carried out this study, which is part of a more comprehensive project about emotions and reactions to the COVID-19 lockdown of children and adolescents with and without high intellectual abilities. The instrument was administered through Google Forms and distributed to parents through social media networks (Facebook and Whatsapp), at the beginning of the lockdown (May–June 2020). The instructions were addressed to the children, and parents could help write the answers, respecting what the children themselves said.

In the case of the group of children with high intellectual abilities, associations working with children with these characteristics were contacted and invited to participate in the study. Parents in both groups were asked to ensure that the children did not consult any sources to answer the questions.

A qualitative methodology was used; the sample is non-probabilistic, by convenience, collected by snowball.

This study was conducted ethically according to the principles of the Declaration of Helsinki. The questionnaire began with informed consent, where parents were notified of the objective of the study and other ethical details such as the confidentiality of the data; after this information, they were asked to give or withhold their consent for children and adolescents to participate in the study. The confidentiality of the data was guaranteed, with no records allowing individual identification of the research participants.

In addition, the ethics committee of the University of Guadalajara was asked for its approval to carry out the research, which was obtained through certification and registration number CI-03820.

### 2.4. Data Analysis

For the qualitative analysis of the responses, the The Analyse Lexicale par Contexte d’un Ensemble de Segments de Texte [ALCESTE] software [36] was used, where the essential information is extracted and the words are grouped by association and proximity, thus differentiating the lexical world [37]. This methodology considers the simultaneous presence of several words in the same sentence. Consequently, classes are identified as semantic fields present in the discourse, represented in dendograms, which are generated by the software [37]. The researcher defines and names each class that makes up the dendogram according to the words that were grouped by the software in each of them.

The purpose of this methodology is to recognize and measure the most robust parts of a piece of writing. The software employs a statistical approach that classifies text sets by means of the chi-square test. It also performs a correspondence factor analysis process. This approach centers on the statistical organization of word sequences that form the sentences within a written composition. It takes into account the simultaneous appearance of various words (nouns, adjectives, and verbs), omitting the analysis of prepositions or conjunctions, among other elements. The objective is to differentiate the most significant lexical groupings.

This methodology has been used in other qualitative studies involving a population with high intellectual ability [25,31]. 

## 3. Results

The results are presented below based on the answers given to the questions used to find out the knowledge and attitudes of children and adolescents regarding the COVID-19 lockdown.

### 3.1. I Believe That the Coronavirus (COVID-19) Is…

**Figure 1:**
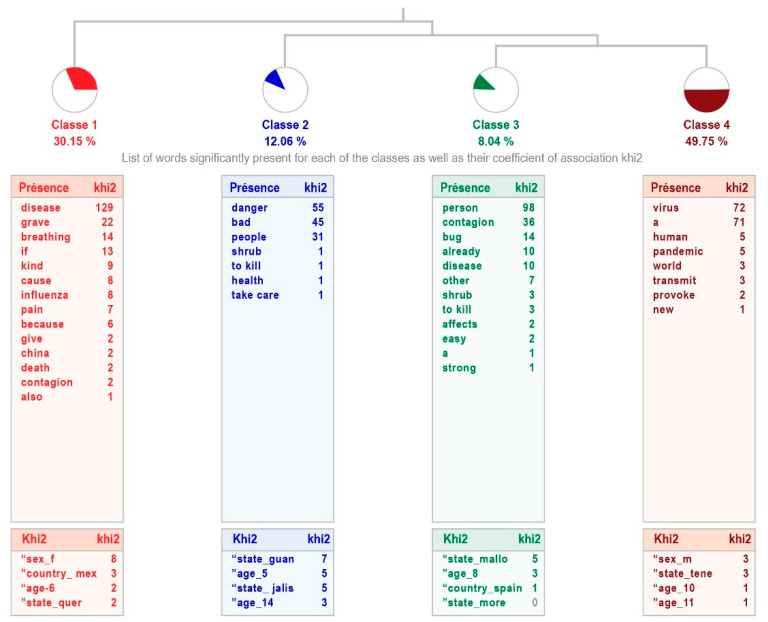
Dendogram showing how children with high intellectual abilities define COVID-19.

The analysis for the group of high-intellectual-ability students shows four classes (“Disease”, “Dangerous virus”, “Possibility of contagion”, and “Virus”), with an average processing relevance, explaining 64% of the answers given. It reveals a tree-like relationship, since the first class “disease” connects with the second one “dangerous virus”, and this one with classes 3 “possibility of contagion” and 4 “virus”, which are linked, the latter class being the most relevant. The combination of these classes indicates that students with high intellectual abilities recognize the coronavirus as a disease caused by a dangerous and easily transmissible virus (see Figure 1).

Table 3 shows the content of the phrases that make up each class, indicating the word with the highest χ^2^.

**Figure 2:**
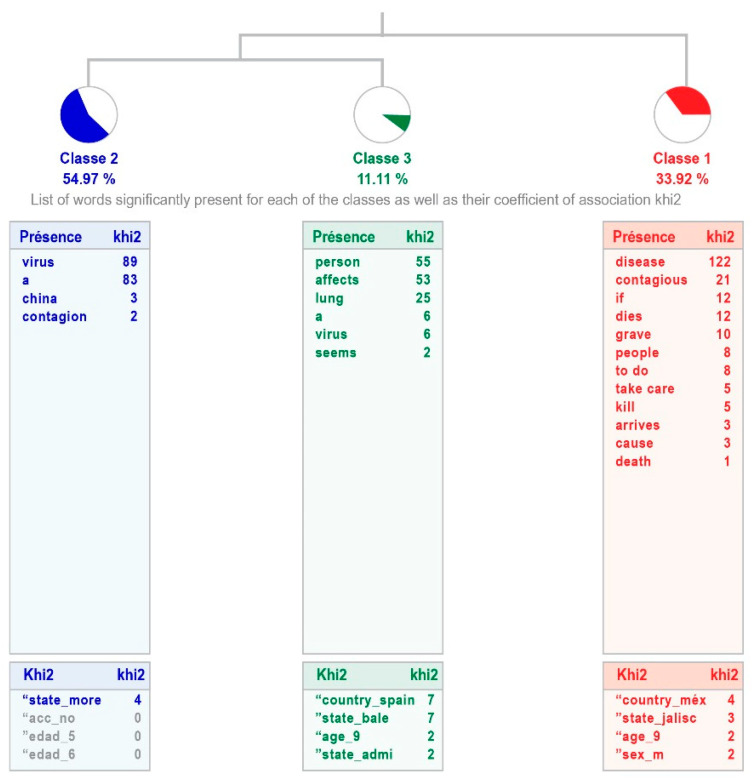
Dendogram showing how children with average intellectual abilities define COVID-19.

The group of children and adolescents without high intellectual abilities show three classes, with a weak processing relevance, explaining 53% of the answers given. The class 1 “Disease” links with classes 2 “virus” and 3 “A virus that affects people”. The most representative is the second class, which groups 54.95% of the text (see Figure 2). In Table 4 is shown the content of the sentences that make up each class, indicating the word with the highest χ^2^.

No significant differences are observed in the two samples. Although the responses of the participants with high intellectual abilities are more varied when explaining why the disease occurs, by including one more class, the explanations of both groups are similar: speaking of disease, produced by a virus, which affects people, explained in more or less detail. The different class in the sample with high intellectual abilities points out one of the aspects of this disease, its contagiousness.

### 3.2. I Know That the Coronavirus (COVID-19) Is Transmitted by…

**Figure 3:**
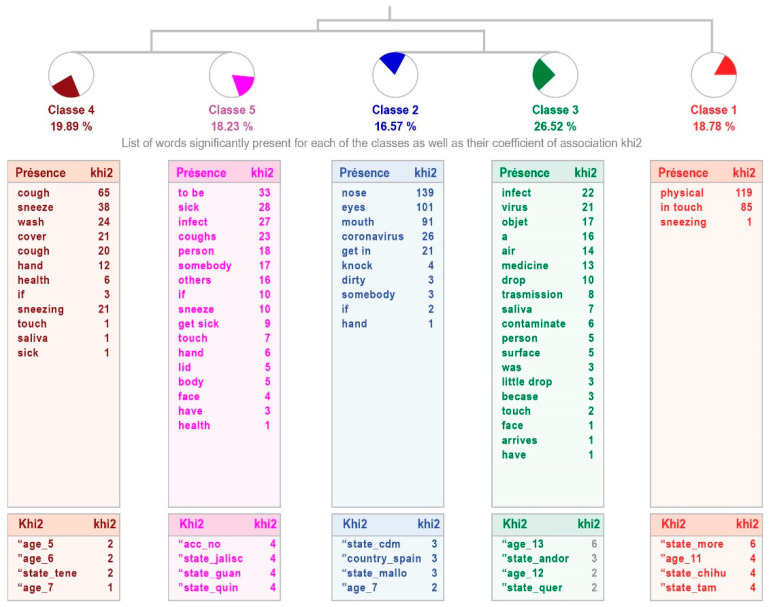
Dendogram showing how children with high intellectual abilities perceive that the coronavirus (COVID-19) is transmitted.

The analysis in the group with high intellectual abilities shows differences in the explanations provided, since the responses are grouped into five classes. The relevance of the process is weak, although it allows 58% of the answers to be ordered. The first class, “Physical contact”, is linked to the classes that come together in pairs: 4 “Infection by contact” and 5 “Person-to-person transmission”, as well as 2 “Infection through areas on the face” and 3 “Airborne infection”. These classes refer to the form of infection by the type, while the first ones refer to the person who can infect. The most representative class is 3, which accounts for 26.52% of the text units analyzed (see Figure 3).

Table 5 shows the content of the phrases that make up each class, indicating the word with the highest χ^2^.

**Figure 4:**
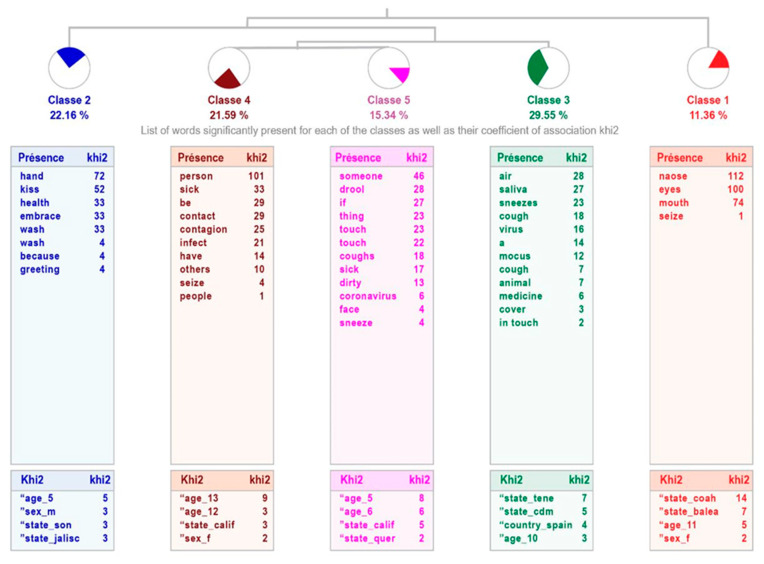
Dendogram showing how children with average intellectual abilities perceive that the coronavirus (COVID-19) is transmitted.

For the sample of the children without high intellectual abilities, the analysis of the way in which the disease is transmitted also results in five classes, although the arrangement differs from the previous one, with different groupings: class 1 “Infection through areas on the face” links with class 2 “Physical contact”, which connects with class 3 “Airborne transmission”; this is related to the tandem formed by classes 4 “Person-to-person transmission” and 5 “Infection by mucus”, which talk about the way it is transmitted. The relevance of the treatment is weak, explaining 54% of text units. The most representative class is 3, grouping 52 text units, that is, 29.55% of them (see Figure 4).

Table 6 shows the content of the phrases that make up each class, indicating the word with the highest χ^2^.

Although the structure differs in both samples, the contents are very similar. Evidently, the pandemic has led to important knowledge of its characteristics and means of transmission, even in the young population.

### 3.3. I Understand That I Should Stay at Home Because…

**Figure 5:**
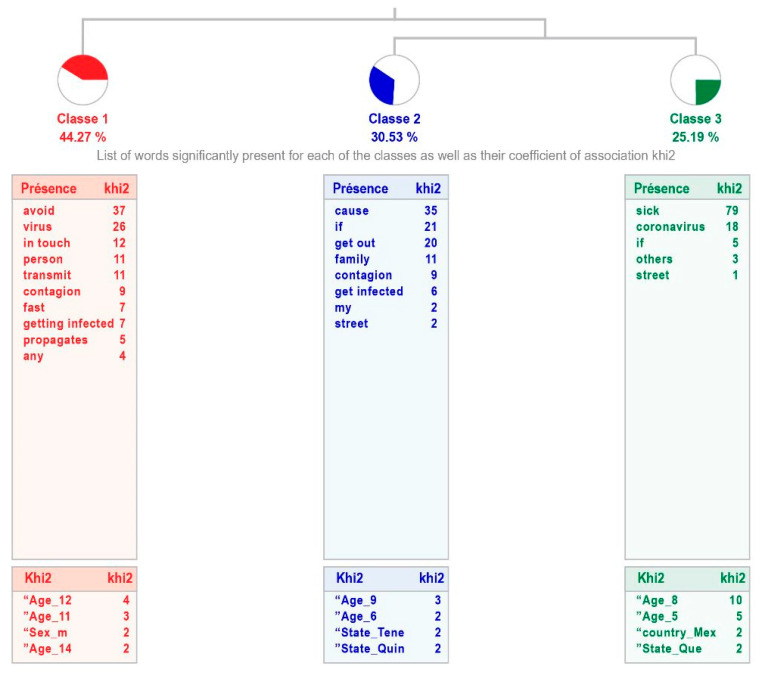
Dendogram showing why children with high intellectual abilities perceive that they should stay at home.

Among all the questions analyzed, in the case of students with high intellectual abilities, this is the one with the weakest treatment relevance, as it classifies 42% of the text units. It generates three classes, the most representative being the first one, “Avoid infection”, which groups 44.27% of the classified text units. This links to the union between classes 2 “Getting infected” and 3 “Getting sick with Coronavirus”. The sample considers that they should stay at home either to avoid spreading the disease (more social sense) or, from a more personal perspective, to prevent infection or not catching the disease (see Figure 5).

Table 7 shows the content of the phrases that make up each class, indicating the word with the highest χ^2^.

**Figure 6:**
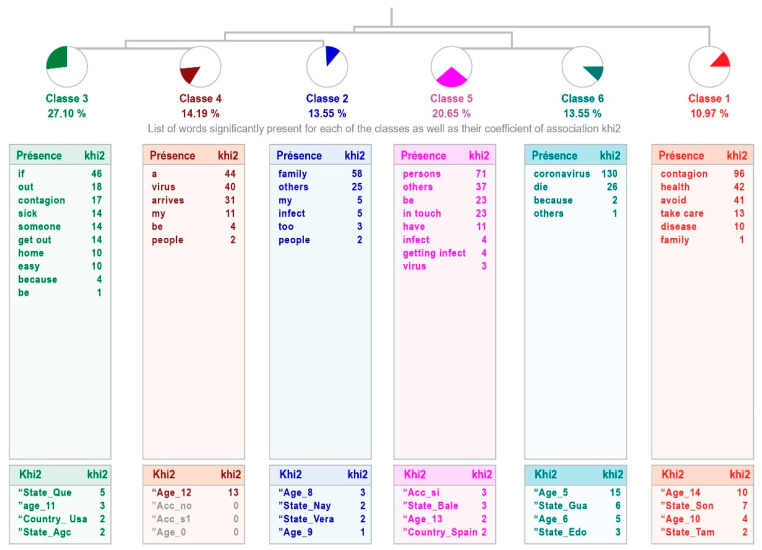
Dendogram showing why children with average intellectual abilities perceive that they should stay at home.

In the sample of students without high intellectual abilities, the responses show greater dispersion, since they are grouped into six classes, but with a treatment relevance that is also weak, although a little higher with 48% of the text units. Class 1 “Avoid infection” connects with the two links, the one between classes 2 “No getting infected and infect others”, 3 “So that they do not infect me”, and 4 “Fear to contagion”, which raises concerns about the risk of spreading the coronavirus to themselves and others, as well as the one between classes 5 “Avoiding infected people” and 6 “Coronavirus infection”, the combination related to prevention (see Figure 6).

Table 8 shows the content of the phrases that make up each class, indicating the word with the highest χ^2^.

The main difference between the two samples is that while the fears of the average-capability participants have a personal perspective (fear of getting sick or infected) and cause them to stay at home, the participants with high intellectual abilities show a more social perspective, which is the fear of infecting others.

## 4. Discussion

The goal of this study was to analyze the knowledge that children and adolescents with and without high intellectual abilities have about the COVID-19 pandemic, examining if there are differences between the two groups, with the intention of assessing whether the lockdown measures might be perceived differently based on their available information.

Regarding the first question (“I believe that the Coronavirus [COVID-19] is…”), which refers to what they understand about the disease, there is clearly good information about it, since they not only know that it is a disease, but that it is caused by a virus. What was found in this study shows that children with and without high intellectual abilities have received information in such a way that they can give a definition about COVID-19, which matches the one provided by the WHO [1]. This demonstrates the great impact that the disease has had on the population, even in children and adolescents, who provided reliable information about COVID-19.

It is inferred that there has been an impact of the information given to the population, as the UNICEF study [38] suggests, which also agrees with the explanation of Dalton et al. [39]: Children and adolescents have gathered information from different sources, which are not limited to their families, but also include the media and social media. It is also important that there are differences between the two samples, for example, the social vision or interest of the students with high intellectual abilities, that is, concern for the speed of contagion worldwide, which corresponds to what Amend et al. [32] mentioned regarding the high emotional perception of this group, in addition to the ability for reasoning and understanding of the phenomenon in this case, COVID-19, and a highly developed social vision. 

Regarding the knowledge that children and adolescents with and without high intellectual abilities have about COVID-19, analyzed through the incomplete sentence “I know that the Coronavirus (COVID-19) is transmitted by…”, they respond that it is transmitted by physical contact, touching the face, through the air, by contact with other people, and from person to person. It can be inferred, as previously mentioned, that the information received and internalized by children and adolescents with and without high intellectual abilities through the different media is in line with that disseminated worldwide or responds to the global reality, as already reported by the WHO [1]. On this point, the means of transmission also correspond to what was reported by Colomé-Hidalgo et al. [40] in adults, regarding the study carried out with the Dominican population, which reflected an acceptable level of knowledge in accordance with the local reality. Similar results were found with adults in Mexico [7,16] and Colombia [41]. In other words, the child and teenage populations, with and without high intellectual abilities, are aware of the means of transmission of COVID-19. Once again, this fact shows the great impact that the disease has had on the population, even on the youngest, who have internalized where the risk factors for transmission are.

Finally, in relation to the understanding of the need to stay at home, as a measure of confinement under the statement “I understand that I must stay at home because…” that children and adolescents with and without high intellectual abilities expressed, the main explanation was to avoid contagion, as a result of the dissemination of information by the WHO [42], as mentioned in the studies conducted by UNICEF [38]. The differences between the groups relate to the social awareness possessed by the children with high intellectual abilities, corresponding to what was described by Albes et al. [43]. These children present a high moral sense of right and wrong, fair and unfair, what should be, and the implications that some facts have on the individual and social level. 

When faced with a new disease such as COVID-19, we observed that both samples handle almost similar information. However, it is important to highlight the social component that stands out in the responses of children and adolescents with high intellectual abilities, reflecting their social sensitivity [33], which is a characteristic of this population, evident on health issues, such as this one. One of the differentiating characteristics between the two groups was in the type of grouping of the classes in the dendograms; for example, in the average ability group, there was more dispersion of the responses, increasing the number of classes.

Children and adolescents have been exposed to numerous sources of information: the media, social media, and of course, their parents or guardians. Likewise, they perceive in adults the concerns and reactions to the crises generated by COVID-19, and they are the ones shaping coping strategies. Therefore, it is important to protect children’s mental health by promoting adequate communication about the disease and its consequences [14,39].

One of the limitations of this study is that, since the questionnaire was applied online, there is no certainty that the parents did not influence the responses of the children and adolescents. Nevertheless, this study has allowed us to deepen the knowledge that both populations (children and adolescents with and without high intellectual abilities) have regarding COVID-19, at a time when the characteristics of the disease, as well as the strategies for dealing with it, were just being learned. Regardless of their intellectual capability, both populations have similar knowledge in general, the most important difference being, however, the social interest shown by students with high intellectual abilities.

Finally, at the national level, as of June 2020, statistics showed that 65% of households were highly concerned about the health consequences of the virus; the reduction in the purchasing power of families affected their ability to access sufficient and nutritious food in some households (42% of households with children are food-insecure in Mexico). This shows that families with children face greater pressure during the pandemic. In terms of education, by the end of the 2019–2020 school year, nine out of ten children were able to continue their education during the lockdown period, and in households with fewer resources, two out of ten were unable to continue their education [44].

Actions in favor of health and physical and emotional well-being that governments, schools, and families carry out will be crucial for mitigating the impact of the pandemic on children and adolescents, regardless of their intellectual capability [25].

## 5. Conclusions

Although children and adolescents with and without high intellectual abilities received information about COVID, their interpretation was similar, according to what was found in this study. Despite this, children and adolescents with high intellectual ability had a greater vision of the health problem, being aware of the global population impact, given their ability to generalize, as well as showing social sensitivity, with concerns about the impact of the pandemic. It is important to consider these differences in regards to emotional support in situations of great impact in terms of health. On the other hand, improving the manner in which information is disseminated across diverse media platforms to mitigate heightened apprehension in times of uncertainty is important, as witnessed during the COVID-19 pandemic.

## Figures and Tables

**Table 1:** Characteristics of participants.

Type of Group	Sex		Age	Country	Type of School	
	Male	Female			Public	Private
High intellectual abilities	204	115	Media = 8.94	Mexico, Spain	190	129
Average intellectual abilities	175	155	Media = 9.04	Mexico, Spain, USA	168	162

**Table 2:** Questions used to determine the knowledge and attitudes of children and adolescents regarding the COVID-19 lockdown.

Item	Description
1	I believe that the Coronavirus (COVID-19) is…
2	I know that Coronavirus (COVID-19) is transmitted by…
3	I understand that I should stay home because…
4	My favorite thing about staying at home during these days is…
5	What bothers me most about staying at home during these days is…
6	I have learned that I can take care of myself by…
7	My parents think that the Coronavirus (COVID-19) is…

**Table 3:** Classes. How children with high intellectual abilities define COVID-19.

High Intellectual Abilities					
Class	Phrases	UCE	%	χ^2^	Word
1	**It is a disease**	**60**	**30.15**		**Disease**
	It is a serious disease.			7	
	It is a serious disease that spreads fast.			7	
	It is a serious disease that can kill you and you can get severe aches. It is like a flu, but stronger.			7	
2	**Dangerous virus**	**24**	**12.06**		**Danger**
	Dangerous and very bad			25	
	It is very bad and has killed many people.			25	
	It is a dangerous virus that is killing many people.			11	
3	**Chance of infection**	**16**	**8.04**		**Person**
	A virus that rapidly and easily infects many people. It is in many countries. Some of the people from infected countries are traveling to Mexico.			30	
	We are not infected so we have to stop doing inappropriate things that aren’t useful and do as much as possible to stay at home and cheer everyone else in our houses and others for a more positive COVID-19			26	
	A virus that kills people and if you don’t wash your hands you can get infected.			16	
4	**Virus**	**99**	**49.75%**		**Virus**
	A virus transmitted by an animal			4	
	An exaggerated virus that spreads all over the world and we can overcome.			4	
	It is a virus that became a pandemic a few days ago.			4	

Note: The words in bold correspond to the words with the highest frequency in the analyses of each classe. The words highlighted in colour correspond to examples of the most frequent words in each classe, according to the Dendogram. Words in red correspond to Classe 1; words in blue correspond to Classe 2; words in green correspond to Classe 3; words in brown correspond to Classe 4.

**Table 4:** Classes. How children with average intellectual abilities define COVID-19.

Average Intellectual Abilities					
Class	Phrases	UCE	%	χ^2^	Words
1	**It is a disease**	**58**	**33.92**		**Disease**
	A very contagious disease and if it’s not taken care of can be serious			14	
	It is a disease that can cause you to die			8	
	A very ugly disease because it is a disease that can kill people.			7	
2	**Virus**	**94**	**54.97**		**Virus**
	It is a virus that developed in China			5	
	A chinese virus			5	
	The Coronavirus is a virus			3	
3	**a virus that affects people**	**19**	**11.11**		**Person**
	A virus that affects elderly persons, people with lung problems, diabetes, children’s immune system prevents them from becoming infected. The infected are traveling to Mexico			10	
	a dangerous virus that not only affects health but also affects the population’s economy and sanity.			10	
	a virus that is not very dangerous because it has become weaker as it comes from other countries and affects the lungs of the elderly.			8	

Note: The words in bold correspond to the denomination of each of the classes. Also, the words highlighted in colour correspond to examples of the most frequent words in each Classe, according to the Dendogram. Words in red correspond to Classe 1; words in blue correspond to Classe 2; words in green correspond to Classe 3.

**Table 5:** Classes. How children with high intellectual abilities perceive that the coronavirus (COVID-19) is transmitted.

High Intellectual Abilities					
Class	Phrases	UCE	%	χ^2^	Word
1	**Physical contact**	**34**	**18.78**		**Physical**
	Physical contact and respiratory tract			23	
	Bats, physical contact, pangolin.			16	
	By physical contact			16	
2	**Infection through areas on the face**	**30**	**16.57**		**Nose**
	If a person has coronavirus and meets someone else, being very close, he can get it through the eyes, nose, ears or mouth.			23	
	Eyes, mouth, nose.			21	
	Flush mouth, nose, eyes, bruises It is ending that is killing a lot of people			21	
3	**Airborne infection**	**48**	**26.52**		**Infection**
	The saliva, airborne because it is alive for a while.			14	
	An airborne virus transmitted by infected things.			13	
	Through the saliva and the sight I heard that on the internet.			11	
4	**Infection by contact**	**36**	**19.89**		**Coughing**
	Handshake, kissing or by not covering properly while sneezing or coughing.			16	
	Handshake and touching			10	
	Not washing the hands and sneezing			10	
5	**Person-to-person transmission**	**33**	**18.24**		**Being**
	By the body of other infected persons.			14	
	With people that travel around the world, if he is sick and you touch him			14	
	When an infected person doesn’t take precautionary measures and somebody is in front of the infected when coughing or sneezing and the other person touches his face, he gets infected and so on.			13	

Note: The words in bold correspond to the denomination of each of the classes. Also, the words highlighted in colour correspond to examples of the most frequent words in each Classe, according to the Dendogram. Words in red correspond to Classe 1; words in blue correspond to Classe 2; words in green correspond to Classe 3, words in brown correspond to Classe 4, words in pink correspond to Classe 5.

**Table 6:** Classes. How children with average intellectual abilities perceive that the coronavirus (COVID-19) is transmitted.

Average Intellectual Abilities					
Class	Phrases	UCE	%	χ^2^	Word
1	**Infection through areas on the face**	**20**	**11.36**		**Nose**
	respiratory tract, eyes, mouth.			35	
	mucous membranes, eyes, mouth and nose			26	
	Eyes, nose and mouth			26	
2	**Physical contact**	**39**	**22.16**		**Hand**
	By kissing, hugging and shaking hands			23	
	Kissing, hugging without washing the hands			23	
	Shaking hands, Kiss, hugs			23	
3	**Airborne transmission**	**52**	**29.55**		**Air**
	it is an airborne virus.			13	
	The air a virus			10	
	Coughing and sneezing.			7	
4	**Person-to-person transmission**	**38**	**21.59**		**Person**
	By being in touch with people that are infected.			12	
	Sick people infect others.			12	
	By being in touch with an infected person.			12	
5	**Infection by mucus**	**27**	**15.34**		**Someone**
	If you are dirty or someone sneezes or coughs in front of you.			25	
	If I touch or suck on something or if someone sick sneezes.			25	
	If someone sneezes or coughs, the Coronavirus Chief jumps and gets to the other person.			19	

Note: The words in bold correspond to the denomination of each of the classes. Also, the words highlighted in colour correspond to examples of the most frequent words in each Classe, according to the Dendogram. Words in red correspond to Classe 1; words in blue correspond to Classe 2; words in green correspond to Classe 3, words in brown correspond to Classe 4, words in pink correspond to Classe 5.

**Table 7:** Classes. Why children with high intellectual abilities perceive that they should stay at home.

High Intellectual Abilities					
Class	Phrases	UCE	%	χ^2^	Word
1	**Avoid infection**	**58**	**44.27**		**Avoid**
	To avoid spreading the virus.			12	
	This way I avoid spreading the virus.			12	
	It is the best way of not spreading the virus.			8	
2	**Getting infected**	**40**	**30.53**		**Because**
	Because if I go out I will get infected.			5	
	nothing may happen to me if I get infected, but then I become a carrier and I can infect my family.			5	
	Because if I go out I could get infected.			5	
3	**Getting sick with Coronavirus**	**33**	**25.19**		**Get sick**
	I could get sick with the coronavirus COVID-19.			15	
	If you go out, the Coronavirus you get sick. And you can spread it to the others. It starts with one and then breaks in halves			11	
	Because of Coronavirus if we go out we can get sick.			9	

Note: The words in bold correspond to the denomination of each of the classes. Also, the words highlighted in colour correspond to examples of the most frequent words in each Classe, according to the Dendogram. Words in red correspond to Classe 1; words in blue correspond to Classe 2; words in green correspond to Classe 3.

**Table 8:** Classes. Why children with average intellectual abilities perceive that they should stay at home.

Average Intellectual Abilities					
Class	Phrases	UCE	%	χ^2^	Word
1	**Avoid infection**	**17**	**10.97**		**Contagion**
	Physical contact and respiratory tract			17	
	This reduces the risk of spreading the virus, slows down and avoids overloading the country’s health care system.			17	
	I must avoid contagion			17	
2	**Not getting infected and infect others**	**21**	**13.55**		**Family**
	Yes, my safety and the ones that I live with my family			21	
	So as not to get infected or infect others.			21	
	Otherwise, I can infect others.			21	
3	**So that they do not infect me**	**42**	**27.10**		**If**
	Because if I go out someone can get me infected.			7	
	Because if we go out someone sick can get us infected			7	
	If I go out someone could get me infected.			5	
4	**Fear to contagion**	**22**	**14.19**		**A**
	I should not get the virus because it can be fatal.			30	
	if I catch the virus I could pass it to my parents and we would have a serious problem.			21	
	it is a very contagious virus.			12	
5	**Avoiding infected people**	**32**	**20.65**		**Person**
	because we don’t have to have contact with other people who could be infected.			18	
	I can get infected by being with other people			17	
	because being in contact with many people makes you more likely to get sick.			11	
6	**Coronavirus infection**	**21**	**13.54**		**Coronavirus**
	I can get coronavirus and die.			35	
	because I can get coronavirus and I can die I have toys.			10	
	because I can’t go out because I get coronavirus.			10	

Note: The words in bold correspond to the denomination of each of the classes. Also, the words highlighted in colour correspond to examples of the most frequent words in each Classe, according to the Dendogram. Words in red correspond to Classe 1; words in blue correspond to Classe 2; words in green correspond to Classe 3, words in brown correspond to Classe 4, words in pink correspond to Classe 5, words in cyan correspond to Classe 6.

## Data Availability

Data can be requested from the corresponding author.
